# Observations on “Congestion”

**Published:** 1864-08

**Authors:** 


CHICAGO
MEDICAL JOURNAL.

VOL. XXI.] AUGUST, 1864. [No. 8

### ORIGINAL COMMUNICATIONS.

### OBSERVATIONS ON "CONGESTION."

By J. A. A.

"What is in a name?" Much—very much. Even life sometimes, when a physician uses it—for with him a name grows to be a "real thing,"—a power which influences and controls, when influence and control poise the nice balance of dangerous disease.

There is scarcely any other name, than this of CONGESTION, which is oftener used by physicians to patients and their friends, or which figures more ominously in Mortality Reports.

The word is a cloak for a multitude of organic sins. The word means, a massing together, a heaping up, a crowding—technically an extraordinary accumulation of blood in a part, with diminution, or total cessation, of its normal motion, "*Stasis of Blood.*" And this, we are told, may be active or passive. And some say that the phrase *active* congestion involves the idea of too much life, whilst the term *passive* recognizes that there is too little life. If this be true, this is a wonderful difference! Somebody says something about loss of contractility, or irritability, or some other *ility* of the capillary parietes as though that meant anything!

In the light of modern science, all these words ending in *ility* are *voces et preterea nihil.* Some one says something about *tonicity*, or some other *icity*, as though that meant something. I pray you, my friend, get rid of the *icities* as well as the *ilities*, they are all shadows and nonsense!

When the great, or simple processes of nature are referred to *properties*, we sin with the alchemists of centuries ago, without the excuse of alchemists of centuries ago. The sin of that ignorance the God of Science winked at, but now commandeth —quite otherwise.

What do we mean by *Congestion?* To understand the stasis of blood—congestion—hyperæmia—or whatever we please to call it—be it " active," or " passive," or neuter, it is necessary to understand why the blood flows in arteries, capillaries, or veins, at all.

My Veratrum Viride friend says, it is because he has not administered his pet specific, and I am quite willing to admit that he not infrequently renders the flow impossible, by its administration. And so he of the Aconite, or Tartar Emetic, or Lobelia persuasion!

Somewhere about 1619, Anno Domini, Harvey demonstrated that the blood passed from the left ventricle of the heart to the aorta, and from that great tube to the arteries, thence to the capillaries, thence to the veins, and thence to the right auricle of the heart, the right ventricle, the lungs, the left auricle, and thence to the left ventricle, and so on again. The result was, the whole medical world began to believe, and much of it still believes, that in the heart are the " issues of life," and in it the fountains of disease. And much of medical practice has been based on controlling this muscular viscus.

Hence the Veratrum Viride man, and the Aconite man, and the Digitalis man, *et id genus omne*, have devoted their collected genius to the great work of controlling the aberrations of this viscus. Opposing the effect—forgetting the cause!

Scriptural physiology is still the best—the *Life* of man is in the *Blood.*

The heart is a necessary organ, a vital organ, but the great changes in which the operations of life essentially consist, take place in the interstices of tissue around the capillaries.

The great force of the circulation is found in the capillary tissue.

This force is the exact measure of the amount of tissue metamorphosis.

When tissue metamorphosis is at its maximum, the rapidity of the local circulation is at its maximum—there is "*determination of blood,*" *hyperœmia*, ultimately *hypertrophy*—or, if formative power be deficient, or there is defective blood, there is suppuration or kakoplastic deposit.

When the blood is deficient in the appropriate elements of nutrition there is necessarily less capillary force. When *poisons* are in it, there is necessarily less capillary force. This, whether you call the force *physical* or *vital.*

When the capillary force is lessened, there is always *Congestion.*

When the capillary force is increased beyond the capacity of the formative force, there is always *Congestion.*

The first is passive, the second active.

——"Facies non omnibus una,

Nec diversa tamen; qualem decet esse sororum."

Somebody has recently grown very facetious over the phrase "*blood-poison.*" Is not somebody aware that all we know of disease is summed up in *blood-poison?* In fact, as well as Scripture, the Life of man is in the Blood—and the Death of man, as immediate correlative, is also to be found in the same all-pervading, all sustaining fluid. *Corpora non agunt nisi soluti*—and no tissue is renovated or healed without healthy blood. There is a world of truth in some of these old maxims.

In passive congestion the blood is charged with constituents which lessen or forbid its affinity for the tissues, or there is mechanical obstruction to its flow.

In active congestion the same event follows from a different cause. Excessive tissue change, generally FEVER, or locally, INFLAMMATION, charges the blood with effete materials, so abundant that their non elimination by the overworked excretories produces the same effect as poisons taken in from without—or the exudation and new formation of the inflammatory process involve mechanical obstruction, just as would a ligature upon the vein or veins beyond. Or both these causes conspire.

Thus the cause of the impaction of blood in any part, or briefly, *congestion*, may be in the part itself, or in the whole circulating mass, or localized upon the proximal or distal side of the part. Mechanically obstructive, or dependent on loss of affinity of the blood for the capillary wall.

Thus the cause of congestion may be anything which impairs the affinity, be it chemical or vital, whatever you choose to call it, of the part involved, for the blood which should permeate it, or, *vice versa*, of the blood for the part—or again, mechanical obstruction.

Every pathologist now recognizes the radical diversity of Portal Venous Congestion and Hepatic Venous Congestion, and at the same time, will admit hepatic stasis of blood from the pressure of tumors or deposits, or the immediate or remote local effects of common "inflammation."

He who now says "Congestion of the Liver," as though that gave the whole story, has advanced but little beyond the "Liver Complaint" of the Fathers.

There is yet another element to be taken into consideration in this discussion—one which the vitalists of the Paine stamp consider the only one, and which humoralists are too much disposed to wholly ignore. This element is the nervous apparatus. Before the true mechanism of nervous action was unfolded by the present writer, the pathologist always had a ready excuse for his ignorance of the real changes which take place in the various processes of "*inflammation*" or "*congestion*," by invoking the benevolent interposition of the "vital properties," as though the phrase meant anything!

Men, accustomed to the ordinary methods of scientific inquiry, wandering into the field of medicine, were amazed at the fossil formulæ piled up to bar further progress. The nervous system, with "vital properties," *ilities* and *icities* in abundance, was the chosen field for bush fighting. The vitalists are the shrewdest of intellectual guerillas. The contest seemed necessarily interminable, but happily it is no longer requisite. The mechanism of nervous action being now well understood we can fully appreciate its influence upon the local changes congestion, inflammation, and kindred modifications of natural processes.

For the nerve fibre or tube simply determines molecular and cell changes in the part, which to a greater or less extent increases or diminishes the affinity, be it physical or vital, of the tissue for the blood. There is no new mechanism supplanting that of health—there is no novelty of process.

Change (molecular), however produced at one extremity of a nerve conductor, manifests itself by change (molecular) at the other extremity of the nerve conductor, (or it may be a second or even further series—"reflex,") increasing or lessening the affinities, or changing the structure involving dilatation or contraction of the contractile parietes of the blood vessels, or modifying nutrition or secretion, (identical physiologic processes,) and so on.

The whole secret of the nervous action in the part is involved in the molecular change produced, be it increase, lessening or mere alteration.

Both pathologically and therapeutically this change is of the highest interest.

Once for all, this change is not an abstract, metaphysical, *spiritual* affair, but a positive, *material* composition and decomposition. Not a mere play upon "*properties*," but a physical change of elements and compounds, as clear and unmistakable as the reactions of a chemical mixture. Whether it be chemical or no, is *not* the question. Congestion may be produced by the nervous action alone. The blush is a temporary congestion from this cause. A jaundice, a diarrhœa, diuresis, diaphoresis, etc., etc., how often are they seen from the same cause!

Not more strange than the riving of a tree by electricity, or the explosion of gunpowder, or mixed oxygen and hydrogen, by the "imponderable" heat.

Congestion is only relieved by the restoration of the affinity —call it physical or vital as you please. It may ultimate in death from immediate impairment of the function of the organ. Witness congestion of the lungs, of the brain, of the kidneys, the effect becomes the cause of death.

It may ultimate in gradual dissolution—chronic disease. Or "inflammation" supervenes, with destructive or conservative processes.

Every organ is an excretory so far as every other organ is concerned. Failure of one in its nutrition, or secretion, which is its correlative, fills a part or the whole of the vascular system with effete substance, which lessens the affinity of the blood for another organ or tissue, and congestion ensues. None more frequently perhaps, than the skin and kidneys.

"Congestion of the brain," "congestion of the lungs," "congestion of the bowels," etc., etc., are oftener the results of morbific causes operating on the skin or kidneys, than from inherent affection of the parts themselves. In this climate the skin and kidneys are especially liable to be the organs at fault. The kidneys are the most important of all excretories—they are the most frequently and readily called into vicarious action. It is safe to say that half at least of the so-called congestions of remote organs, brain, lungs, etc., are the result of failure of the kidneys or skin in their eliminatory functions.

Hence blood-poisoning, a phrase sneered at by sciolists, by vitalists and worshipers of Paine.

Alvine excretions may fail, but so long as the kidneys are perfect in the performance of their function, remote organs, and the system in general, escape harm. They are the true safety valves.

True therapeutics consist in elimination—restoration of normal conditions, and provision for *renewal*.

Not only the vascular system, but the nervous system, and last not least, the nutrient apparatus must be attended to by the therapeutists.

Topographical—"specific" nosology and nostrum making, regular and irregular, have had their day. PHYSIOLOGY, *otherwise* NATURE, is now in the ascendant.

